# A chromosomal-level genome assembly of *Nadezhdiella cantori* (Hope, 1843) (Coleoptera: Cerambycidae)

**DOI:** 10.1038/s41597-026-07420-y

**Published:** 2026-05-12

**Authors:** Jianfeng Jin, Maolin Ye, Hanmo Zhang, Fang Zheng, Yanan Li, Bin Yu, Shengli Jing

**Affiliations:** 1 College of Life Sciences, Xinyang Normal University, Xinyang, 464000 China; 2 College of Plant Protection, South China Agricultural University, Guangzhou, 510600 China

**Keywords:** Genome, Genomics

## Abstract

*Nadezhdiella cantori* (Coleoptera: Cerambycidae) is a wood-boring pest native to Southeast Asia that attacks citrus trees, with larvae boring into trunks and branches and often causing tree mortality. Nevertheless, the absence of a high-quality genome has hindered ecological research and the development of effective pest management for *N. cantori*. In this study, we report the assembly of a chromosome-level genome for *N. cantori*, generated by integrating PacBio, Illumina, and Hi-C sequencing data. The assembled genome is 737.74 Mb in size, comprising 30 scaffolds and 943 contigs, with scaffold and contig N50 values of 87.91 Mb and 1.33 Mb, respectively. In total, 97.88% (722.07 Mb) of the genome was assigned to 10 chromosomes. BUSCO analysis using the insecta_odb10 dataset (n = 1,367) revealed the genome assembly completeness of 99.3%, including 98.1% single-copy and 1.2% duplicated BUSCOs. We identified repetitive elements constituting 61.20% (451.48 Mb) of the genome and predicted 14,883 protein-coding genes. This genome serves as a valuable resource for *N. cantori*’s adaptations and developing wood-boring pest control.

## Background & Summary

Longhorned beetles (Coleoptera: Cerambycidae) represent a diverse and economically significant insect family^1–3^. Boasting approximately 4,000 genera and over 35,000 described species, Cerambycidae represents one of the largest beetle families^1,4,5^. These beetles exhibit a near-global distribution, occurring on all continents except Antarctica, and inhabit environments ranging from sea level to montane elevations exceeding 4,000 meters^6,7^. Cerambycids are exclusively phytophagous, with larvae primarily developing as internal feeders within living or dead plant tissues; however, many species also exhibit external root-feeding behavior^8,9^. Cerambycid beetles play crucial ecological roles, particularly in forest ecosystems where they are major decomposers of dead plant matter. They also serve as pollinators for numerous herbaceous plants, shrubs, and trees, and constitute an important food source for many vertebrates^10,11^. Conversely, several cerambycid species are significant pests in agriculture, horticulture, and forestry. Some species also vector harmful plant pathogens, including nematodes and fungi^12,13^. Furthermore, the progressive globalization of trade and travel in recent decades has facilitated the accidental introduction of cerambycid pests into new regions, often with severe ecological and economic consequences^14,15^.

The family Cerambycidae is a charismatic beetle family popular with collectors for centuries, exhibiting remarkable biological and morphological diversity^8^. Its members display striking biological and morphological diversity, with body sizes ranging from just a few millimeters to over 17 centimeters^8^. While many are nocturnal and cryptically colored, others are diurnal and display spectacular mimicry of hymenopteran forms (e.g., bees, wasps, ants) and behaviors^16^. Cerambycids are associated with a vast array of plant hosts, including grasses, bamboo, and conifers^17^. Their larvae exploit nearly all parts of host trees, from roots and trunks to branches, leaves, and seeds^18^. Among these species, *Nadezhdiella cantori* (Hope, 1843) is native to Southeast Asia and is widely distributed across southern China, and Thailand^19,20^. Its primary hosts are citrus trees, including *Citrus tangerina*, *C. limon*, *C. sinensis*, *C. aurantifolia*, *C. limonia*, and *C. maxima*^21^. This species is one of the most abundant and destructive cerambycids in China^19^. The larvae bore into the inner bark and sapwood of living trees’ trunks and main branches, weakening them and making them prone to wind breakage; heavy infestations can ultimately kill the tree^19^. High-quality genomes of Cerambycidae offer key insights into their biology and ecology while facilitating the development of effective pest management strategies. Genomic analysis of the chromosome-level *Anoplophora glabripennis* genome revealed horizontally acquired glycoside hydrolases that likely enhance its ability to extract nutrients from nutrient-poor, recalcitrant woody tissues^22,23^. Recently, chromosome-level genome assemblies have been generated for four additional ecologically and economically important xylophagous longhorned beetles^24–27^, including *Monochamus alternatus*, *Monochamus saltuarius*, *Arhopalus rusticus*, and *M. scutellatus*. These genomes provide valuable resources for exploring the genomic basis of conifer feeding and adaptive traits associated with forest environments. However, no assembled genome is currently available for *N. cantori*, and this lack has hindered studies of its biology as well as the development of effective control strategies.

In this study, we present a chromosome-level genome of *N. cantori*, assembled using PacBio HiFi, Illumina, and Hi-C data. We comprehensively annotated repetitive elements, non-coding RNAs, and protein-coding genes. This high-quality reference genome offers a valuable resource for studying the ecology, genetics, and evolutionary history of *N. cantori*.

## Methods

### Sample collection and sequencing

A single female specimen of *N. cantori* was collected from Nanchong Farm, Hekou Town, Yangchun City, Yangjiang, Guangdong Province, China (21.9353°N, 111.5789°E; 27 m a.s.l.) on 3 March 2025, and was used for sequencing. Muscle tissue was collected from the pronotum region of this individual. To minimize potential external contaminants, the harvested tissue was thoroughly washed in phosphate-buffered saline for 10 minutes. Following this cleaning step, the tissue sample was flash-frozen in liquid nitrogen for 20 minutes to preserve nucleic acid integrity and was subsequently stored at −80°C in the laboratory until the initiation of sequencing procedures.

Genomic DNA was extracted with the DNeasy Blood & Tissue Kit (Qiagen), and RNA was isolated using TRIzol reagent, following the respective manufacturers’ protocols. PCR-free short-read libraries were constructed with the Illumina TruSeq DNA PCR-Free Kit. The Hi-C protocol commenced with the digestion of genomic DNA using MboI restriction enzyme, followed by ligation using T4 DNA ligase to generate chimeric junctions^28^. Subsequently, these junctions were enriched and physically sheared to obtain fragments of the target length (350 bp) for downstream sequencing analyses. Using these chimeric fragments, paired-end sequencing libraries were constructed, which capture the original cross-linked long-range physical interactions. For short-read sequencing, we employed the Illumina NovaSeq. 6000 platform. Long-read sequencing utilized a 20 kb SMRTbell library prepared with the PacBio SMRTbell Express Template Prep Kit 2.0 and sequenced in HiFi mode on a PacBio Sequel II system. Berry Genomics (Beijing, China) handled library preparation and sequencing. Sequencing yielded a total of 101.04 Gb of data, comprising 9.89 Gb of PacBio HiFi reads (13.41 × coverage), 34.18 Gb of Illumina short reads (46.33 × ), 16.73 Gb of transcriptome reads, and 40.24 Gb of Hi-C data (54.55 × ) (Table 1).

**Table 1:** Statistics of the sequencing data used for genome assembly.

Libraries	Insert sizes (bp)	Clean data (Gb)	Sequencing coverage (x)
Illumina	350	34.18	46.33
PacBio HiFi	20 Kb	9.89	13.41
Hi-C	350	40.24	54.55
RNA	350	16.73	—

### Genome assembly

**Fig. 1:**
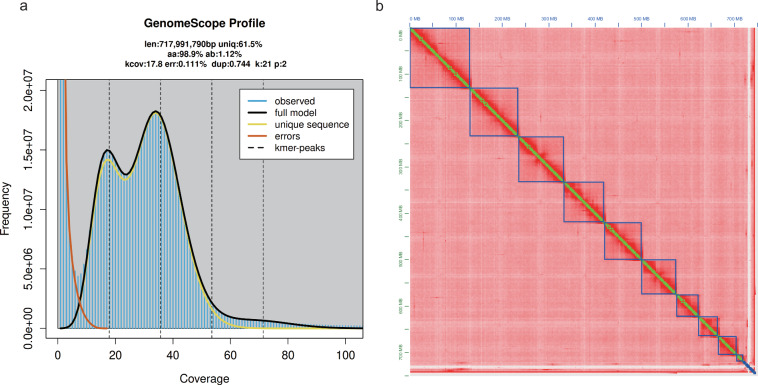
Genome size estimation and chromosomal heatmap of *Nadezhdiella cantori*. (**a**) Genome size estimated using GenomeScope. (**b**) Assembled chromosomes are shown in blue, with green borders indicating contig junctions within scaffolds.

Quality control of raw Illumina reads utilized BBTools v38.82^29^: clumpify.sh removed duplicates, followed by bbduk.sh for adapter trimming and strict filtering (discard Q < 20 or > 5 Ns; trim poly-A/G/C tails > 10 bp; correct overlapping pairs). A GenomeScope v2.0^30^ survey on the processed reads estimated *N. cantori* genome size, heterozygosity, and repetitive sequence content. Genome size was estimated at 715.93–717.99 Mb, of which 275.66–276.46 Mb (38.50%) consisted of repetitive sequences. Heterozygosity was high, estimated at 1.11–1.14% (Fig. 1a).

**Fig. 2:**
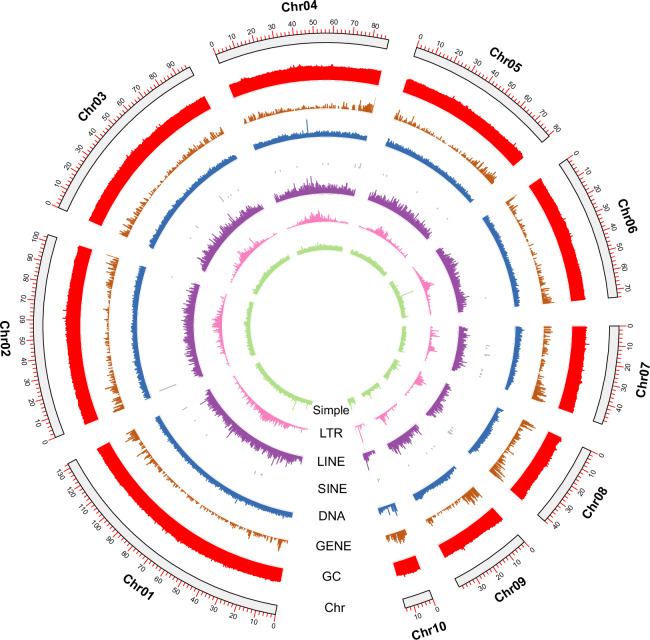
The circular plot of the *Nadezhdiella cantori* genome displays multiple genomic features, arranged from outermost to innermost: (1) chromosome length, (2) GC content, (3) gene density, and (4) repetitive elements, including DNA transposons, SINEs, LINEs, LTRs, and simple repeats. The plot uses a window size of 100,000 bp.

We assembled PacBio HiFi reads into an initial genome using Hifiasm v0.19.8^31^ (default parameters). Heterozygosity was reduced by purging haplotigs with Purge_Dups v1.2.5^32^ (-s 70 haplotype cutoff). Hi-C reads were subjected to quality control and subsequently mapped to the assembly using Juicer v1.6.2^33^. The resulting contact maps were scaffolded into chromosome-scale assemblies using 3D-DNA v180922^34^. The final assembly underwent manual verification and correction in Juicebox v1.11.0^33^ to resolve misjoins and orientation errors. Potential contaminants were identified by screening the assembly against the NCBI nucleotide database using MMseqs. 2 v11.1^35^. Vector contamination was further assessed with BLASTN (BLAST + v2.11.0)^36^ against the UniVec database, and sequences showing > 90% identity to UniVec entries were classified as contaminants. The genome assembly has been submitted to the NCBI genome database for review. A total of 14 contaminant sequences, including one fungal and 13 firmicutes, were identified and removed. The final chromosome-level assembly of *N. cantori* spans 737.74 Mb and consists of 30 scaffolds and 943 contigs, closely matching the genome size estimated (717.99 Mb) from the initial k-mer analysis. The final assembly achieved chromosome-level continuity, with scaffold and contig N50 values of 87.91 Mb and 1.33 Mb, respectively (Table 2). Additionally, the assembly anchored 97.88% of the genome (722.07 Mb) to 10 chromosomes, which range in length from 14.44 Mb to 132.43 Mb (Table 3; Figs. 1,2). Chromosomes were named in descending order of length, with the longest designated as chromosome 1 (Table 3). The assembly demonstrates high continuity and structural integrity, with a BUSCO completeness score of 99.3% and strong chromosomal-level organization.

**Table 2:** Genome assembly statistics for *Nadezhdiella cantori*.

Assembly	Total length (Mb)	Number scaffolds/contigs (chromosomes)	Scaffold/contig N50 length (Mb)	GC (%)		BUSCO (n = 1,367) (%)			
					C	S	D	F	M
Hifiasm	892.28	2168/2168	1.14/1.14	33.52	99.5	88.0	11.5	0.1	0.4
Purge_Dups	745.50	976/1001	1.34/1.33	33.29	99.3	98.1	1.2	0.1	0.6
3D-DNA	745.69	187/1134 (10)	87.92/1.32	33.29	99.3	98.1	1.2	0.1	0.6
Final	737.74	30/943 (10)	87.91/1.33	33.29	99.3	98.1	1.2	0.1	0.6

C: complete BUSCOs; S: Complete and single-copy BUSCOs; D: complete and duplicated BUSCOs; F: fragmented BUSCOs; M: missing BUSCOs.

**Table 3:** Statistics for chromosome sequence length.

Chromosome ID	Sequence Length (Mb)
Chr01	132.43
Chr02	103.31
Chr03	97.70
Chr04	87.90
Chr05	80.56
Chr06	74.05
Chr07	49.92
Chr08	42.04
Chr09	39.71
Chr10	14.44

### Genome annotation

Repetitive elements were identified through de novo annotation using RepeatModeler v2.0.4^37^ with the -LTRStruct option enabled to improve detection of LTR retrotransposons. The resulting repeat library was merged with curated sequences from RepBase-20230909^38^ and Dfam v3.5^39^ to build a custom database for downstream analyses. Repetitive sequences were then annotated and masked with RepeatMasker v4.1.2^40^, which aligns the genome against this integrated database. The analysis identified 451.48 Mb, accounting for 61.20% of the *N. cantori* genome assembly, as repetitive sequences. These were classified into unclassified elements (39.66%), DNA transposons (13.38%), LINEs (3.66%), LTR retrotransposons (3.17%), and other repeat types (Table 4).

**Table 4:** Genome assembly and annotation statistics of *Nadezhdiella cantori*.

Characteristics	*N. cantori*
Genome assembly	
Genome Size (Mb)	737.74
Number of scaffolds	30
Number of chromosomes	10
Scaffold N50 length (Mb)	87.91
Contig N50 length (Mb)	1.33
GC (%)	33.29
BUSCO completeness (%)	99.3
Protein-coding genes	
Number	14,883
Mean gene length (bp)	12,801.1
BUSCO completeness (%)	97.7
Repetitive elements	
Size (Mb)	451.48 (61.20%)
DNA transposons (Mb)	99.13 (13.38%)
LINEs (Mb)	27.15 (3.66%)
LTRs (Mb)	23.50 (3.17%)
Unclassified (Mb)	292.62 (39.66%)
ncRNA	
Number of ncRNA	756
tRNA	388
rRNA	28
miRNA	93
snRNA	103
ribozyme	2
Function annotation	
Number of genes matching Uniprot records	14,326
Number of genes with InterProScan annotations	12,358
Number of genes with eggNOG annotations	13,684
Number of genes with COG Functional Categories combining InterProScan and eggNOG results	12,768
Number of genes with GO items combining InterProScan and eggNOG results	10,958
Number of genes with KEGG pathways items combining InterProScan and eggNOG results	5,228

Using Infernal v1.1.4^41^ with the Rfam v14.10^42^ database for ncRNA identification and tRNAscan-SE v2.0.9^43^ for tRNA detection, we identified a diverse ncRNA repertoire in *N. cantori*. We identified 756 ncRNAs, comprising 388 tRNAs, 28 rRNAs, 93 microRNAs, 103 sRNAs, and other RNA types (Table 4).

Protein-coding genes in *N. cantori* were annotated using MAKER v3.01.03^44^, which integrated homology-based evidence, de novo predictions, and transcriptome alignments to generate high-confidence gene models. Transcriptome reads were aligned to the genome with HISAT2 v2.2.1^45^ and assembled into transcripts using StringTie v2.1.6^46^. For ab initio gene prediction, we employed BRAKER v2.1.6^47^, which integrates GeneMark-ES/ET/EP v4.68_lic^48^ and AUGUSTUS v3.4.0^49^. These tools were automatically trained using transcriptome alignments and conserved protein sequences from the OrthoDB v11 database^50^. In addition, gene prediction employed GeMoMa v1.9^51^, utilizing protein homology information from five reference insects: *Drosophila melanogaster* (GCA_000001215.4)^52^, *Apis mellifera* (GCA_003254395.2)^53^, *Bombyx mori* (GCA_030269925.2)^54^, *Aromia moschata* (GCA_029963805.1)^55^, and *Tribolium castaneum* (GCA_031307605.1)^56^ (Table 5). We annotated 14,883 protein-coding genes in *N. cantori* (Table 4), with an average gene length of 12.8 kb. The typical gene structure includes 5.7 exons (average length: 315.7 bp), 4.6 introns (average length: 2,428.2 bp), and 5.5 coding sequence (CDS) segments (average length: 266.4 bp). Gene prediction quality was evaluated using BUSCO (Insecta_odb10; n = 1,367), which revealed 97.6% completeness (1,334 BUSCOs), including 96.2% single-copy (1,315), 1.4% duplicated (19), 1.6% fragmented (22), and 0.8% missing (11). This high level of completeness confirms the reliability of the annotation.

**Table 5:** Species taxonomic information and accession code of all samples used in this study.

Species	Order	Family	Source
*Tribolium castaneum*	Coleoptera	Tenebrionidae	NCBI (GCA_031307605.1)
*Aromia moschata*	Coleoptera	Cerambycidae	NCBI (GCA_965642075.1)
*Apis mellifera*	Hymenoptera	Apidae	NCBI (GCA_003254395.2)
*Bombyx mori*	Lepidoptera	Bombycidae	NCBI (GCA_030269925.2)
*Drosophila melanogaster*	Diptera	Drosophilidae	NCBI (GCA_000001215.4)

Predicted proteins were functionally annotated using DIAMOND v2.0.11.1^57^ for homology searches against the UniProtKB/Swiss-Prot database. Additionally, eggNOG-mapper v2.0.1^58^ and InterProScan 5.53-87.0^59^ were employed to assign Gene Ontology (GO) terms, identify KEGG and Reactome pathways, and annotate protein domains. InterProScan analysis integrated data from five databases: Pfam^60^, SMART^61^, Superfamily^62^, Gene3D^63^, and CDD^64^. The outputs from these resources were integrated to generate comprehensive gene function predictions. In total, 14,326 genes were annotated with UniProt entries, of which 10,958 were assigned GO terms, 5,228 were mapped to KEGG pathways, 3,030 were linked to EC numbers, and 12,768 were classified into COG. The distribution of repetitive elements, GC content, and gene density across all chromosomes was visualized using TBtools^65^.

## Data Records

The sequencing data generated in this study are available under the following National Center for Biotechnology Information (NCBI) SRA accession numbers: Hi-C data (SRR34376234)^66^, transcriptome reads (SRR34376235)^67^, Illumina short reads (SRR34376236)^68^, and PacBio HiFi long reads (SRR34376237)^69^. The final genome assembly is available under NCBI accession GCA_051225655.1^70^. Genome annotation data, including repetitive elements, gene structure predictions, and functional annotations, have been deposited in figshare^71^.

## Technical Validation

Genome assembly quality was evaluated using two complementary approaches. First, we evaluated assembly completeness with BUSCO v5.0.4^72^, utilizing the Insecta reference set (n = 1367). The assembly demonstrated a high BUSCO completeness of 99.3%, with 98.1% of genes present as single copies, 1.2% duplicated, 0.1% fragmented, and only 0.6% missing. Second, accuracy was assessed through read-mapping rates, with PacBio and Illumina reads aligned using Minimap2 v2.23^73^ and SAMtools v1.9^74^, respectively, with the resulting mapping rates serving as a key metric of accuracy. The assembly exhibited high mapping rates for both PacBio (99.16%) and Illumina (97.49%) reads. Together, these complementary analyses confirm that our genome assemblies are of high quality.

## Data Availability

The dataset presented here is novel and has not been published previously. The authors declare no conflicts of interest. The raw sequencing data and assembled genome for *Nadezhdiella cantori* have been deposited in the NCBI under BioProject accession PRJNA1274636. Furthermore, comprehensive annotation files, encompassing repetitive elements, gene models, and functional annotations, are publicly accessible via Figshare at 10.6084/m9.figshare.29546459.

## Code availability

The scripts used for genome assembly and annotation in this article were uploaded to figshare^71^. All commands and pipelines used in data processing were executed according to the manual and protocols of the corresponding bioinformatic software.
